# p53 expression in squamous dysplasia associated with carcinoma of the oesophagus: evidence for field carcinogenesis

**DOI:** 10.1054/bjoc.2000.1443

**Published:** 2000-10

**Authors:** M Yasuda, H Kuwano, M Watanabe, Y Toh, S Ohno, K Sugimachi

**Affiliations:** Department of Surgery, Fukuoka Dental College Hospital, 2–15–1 Tamura, Sawara-ku, Fukuoka, 814–0193; First Department of Surgery, Gunma University School of Medicine, 3–39–15 Showamachi, Maebashi, 371–8511; Second Department of Surgery, Faculty of Medicine, Kyushu University, 3–1–1 Maidashi, Higashi-ku, Fukuoka, 812–8582, Japan

**Keywords:** dysplasia, oesophagus, p53, immunohistochemistry, field carcinogenesis, prognosis

## Abstract

Squamous epithelial dysplasia is often observed multifocally in the cancerous oesophagus and is presumably considered to be a pre-cancerous lesion. A mutation of the p53 tumour suppressor gene is commonly identified in oesophageal cancer and dysplasia. p53 mutations can be anticipated immunohistochemically. In order to confirm the biological and clinical significance of p53 expressions in oesophageal field carcinogenesis, immunostaining for p53 in cancerous and multifocal precancerous lesions from resected human oesophagus was systematically investigated, while paying special attention to the contiguity of these lesions. Lesions expressing p53 were detected in 46.5% (20 of 43 lesions) of the invasive carcinoma, and in 51.0% (46 of 90 lesions) of the carcinoma in situ, and in 51.4% (92 of 179 lesions) of the dysplasia. Next, the p53 expression in dysplasia was compared with that in carcinoma for the same case. 37 of 39 (94.8%) dysplasias contiguous to p53-positive carcinomas also expressed p53 (*P* < 0.0001). On the other hand, the isolated dysplasias without contiguity to p53-positive carcinomas, only expressed p53 protein in 44.0% (11 of 25 lesions). No significant correlations were found between the p53 staining and either the clinicopathological features or prognosis. Discordant p53 alterations, such as those seen in cancerous and isolated precancerous lesions, may thus demonstrate further evidence for a multicentric or field carcinogenesis of the human oesophagus. © 2000 Cancer Research Campaign


*British Journal of Cancer* (2000) **83**(8), 1033–1038
© 2000 Cancer Research Campaign
doi: 10.1054/ bjoc.2000.1443, available online at http://www.idealibrary.com on IDEAL®

### p53 expression in squamous dysplasia associated with carcinoma of the oesophagus: evidence for field carcinogenesis

**M Yasuda[1], H Kuwano[2], M Watanabe[3], Y Toh[3], S Ohno[3] and K Sugimachi[3]**

[1]Department of Surgery, Fukuoka Dental College Hospital, 2–15–1 Tamura, Sawara-ku, Fukuoka 814–0193; [2]First Department of Surgery, Gunma University School of Medicine, 3–39–15 Showamachi Maebashi 371–8511; [3]Second Department of Surgery, Faculty of Medicine, Kyushu University, 3–1–1 Maidashi, Higashi-ku, Fukuoka 812–8582, Japan

**Summary** Squamous epithelial dysplasia is often observed multifocally in the cancerous oesophagus and is presumably considered to be a pre-cancerous lesion. A mutation of the p53 tumour suppressor gene is commonly identified in oesophageal cancer and dysplasia. p53 mutations can be anticipated immunohistochemically. In order to confirm the biological and clinical significance of p53 expressions in oesophageal field carcinogenesis, immunostaining for p53 in cancerous and multifocal precancerous lesions from resected human oesophagus was systematically investigated, while paying special attention to the contiguity of these lesions. Lesions expressing p53 were detected in 46.5% (20 of 43 lesions) of the invasive carcinoma, and in 51.0% (46 of 90 lesions) of the carcinoma in situ, and in 51.4% (92 of 179 lesions) of the dysplasia. Next, the p53 expression in dysplasia was compared with that in carcinoma for the same case. 37 of 39 (94.8%) dysplasias contiguous to p53-positive carcinomas also expressed p53 ($P < 0.0001$). On the other hand, the isolated dysplasias without contiguity to p53-positive carcinomas, only expressed p53 protein in 44.0% (11 of 25 lesions). No significant correlations were found between the p53 staining and either the clinicopathological features or prognosis. Discordant p53 alterations, such as those seen in cancerous and isolated precancerous lesions, may thus demonstrate further evidence for a multicentric or field carcinogenesis of the human oesophagus.
© 2000 Cancer Research Campaign

**Keywords:** dysplasia; oesophagus; p53; immunohistochemistry; field carcinogenesis; prognosis

A mutation of the p53 tumour suppresser gene is the most common genetic abnormality in solid human malignancies (Hollstein et al, 1991). p53 is a transcription factor that regulates cell growth through a cell-cycle checkpoint, which then insures appropriate cell replication and division. Mutant p53 protein changes its conformation and lacks both DNA binding capability and suppressor activity for cell growth. The mutant p53 protein has a prolonged half-life and thus the detection of p53 protein by immunohistochemistry may indicate the presence of p53 mutations (Rogel et al, 1985; Hall and Lane, 1994). Recent studies demonstrated a good correlation in oesophageal carcinoma between immunohistochemical p53 protein accumulation and the presence of p53 gene mutations (Bennett et al, 1991; Wagata et al, 1993; Gao et al, 1994). The timing of such p53 alterations on carcinogenesis and progression may vary among tumours. In oesophageal cancer, the p53 mutational events occur at an early stage of carcinogenesis, including dysplasia which could be considered precancerous lesions of oesophageal cancer (Bennett et al, 1992; Wang et al, 1993; Gao et al, 1994).

Squamous epithelial dysplasia is frequently encountered in the oesophagus with squamous cell carcinoma and is often observed multifocally. We have previously performed serial histopathological investigations for oesophageal carcinomatous lesions, and a close relationship was observed between dysplasia and carcinoma based on the continuity of dysplastic lesions to the carcinoma and the multiplicity of these lesions. Many dysplastic lesions were located separately from the carcinoma (Nagamatsu et al, 1992). In addition, in the cases with dysplastic lesions, the multiplicity of squamous cell carcinoma and the intra-epithelial spread of the main lesion were also more frequently seen (Kuwano et al, 1993; Morita et al, 1994). We also performed a cytophotometric DNA analysis of chemically induced oesophageal carcinoma in rats (Koga et al, 1988) and immunohistochemical detection of proliferating cell nuclear antigen for resected human oesophageal specimens (Koga et al, 1996), and demonstrated that the biological nature of dysplasia might well be as serious a lesion as cancer itself. In view of these results, multifocal dysplastic lesions may occur independently, some of which may develop and progress to carcinoma, thus suggesting the concept of 'field carcinogenesis'.

There have been some reports about the p53 accumulation or mutational status on the 'cancerization field' of oesophageal carcinoma. p53 alterations of carcinoma and nearby dysplasia were identical on resected specimens (Vijeyasingam et al, 1994; Shi et al, 1999). In contrast, discordant mutations were found among multifocal precancerous lesions obtained from biopsy samples of symptom-free subjects in China (Wang et al, 1996). Little information is available regarding a systematic characterization concerning the p53 mutational status of the multifocal cancerous and precancerous lesions on the cancerization field.

Recently, many reports on the prognostic value of p53 mutational status in oesophageal cancer have been published (Sarbia et al, 1994; Lam et al, 1997; Ikeda et al, 1999; Kanamoto

*Received 2 December 1999*
*Revised 5 June 2000*
*Accepted 11 July 2000*

*Correspondence to:* M Yasuda et al, 1999; Kobayashi et al, 1999; Shimada et al, 1999; Wang et al, 1999). The interpretation of these results, however, still remains controversial. In the current study, the immunostaining of accumulated p53 protein was performed for a large number of sections divided from the resected human oesophageal specimens, paying special attention to the continuity of multifocal dysplastic lesions to a carcinoma within individual oesophageal cancer. Moreover, the value of p53 expression as a prognostic indicator of long-term survival in patients of oesophageal carcinoma with dysplasia was examined. The purpose of this report is to confirm the biological and clinical significance of p53 alterations in oesophageal field carcinogenesis.

### MATERIALS AND METHODS

### Patients and clinical outcome

Four hundred and twenty three patients with primary oesophageal carcinoma underwent an oesophageal resection in the Second Department of Surgery, Kyushu University, Fukuoka, Japan, from January 1982 to December 1993. Among these cases, excluding 287 patients with adenocarcinoma and those undergoing preoperative treatment, we reviewed 136 who had undergone no preoperative treatment.

Formalin-fixed paraffin-embedded blocks of the entire resected oesophagus were prepared from 5 mm-wide step-sectioned specimens and were stained with haematoxylin and eosin. The number of various lesions was counted by a histological diagram and their extent was mapped in two dimensions. Among 136 cases of oesophageal squamous cell carcinoma, excluding 101 cases without dysplasia, 35 cases with squamous dysplasia were selected.

The clinicopathological features of these 35 patients were reported according to the pathological tumour-node-metastasis (pTNM) classification of the International Union Against Cancer (Sabin and Wittekind, 1997). The patients were followed up with systemic examinations. The mean follow-up period was 53.0 months (range 0.7–111.5 months).

### Histopathology and tissue selection

For the histological diagnosis of squamous epithelial dysplasia, we used the criteria defined by the World Health Organization's International Histological Classification of Tumours in 1990 (Watanabe et al, 1990). According to these criteria, mild dysplasia means lesions in which atypical nuclei are limited to the basal zone and there is an evidence of cytoplasmic maturation superficially. With increasing grades of dysplasia, a progressive increase in the proportion of atypical basal cells is observed, until finally the entire thickness of the epithelium is replaced. Therefore, moderate dysplasia was diagnosed when an intra-epithelial lesion similar to mild dysplasia with an atypical proliferative zone expanding up to one-half the thickness of the epithelium was found. Severe dysplasia was defined as an intra-epithelial lesion with an atypical proliferative zone encompassing up to three-quarters of the epithelium. Carcinoma in situ was diagnosed when atypical immature cells occupied either the whole or almost the whole thickness of the epithelium but did not invade the basement membrane below the basal zone.

Next, special attention was paid to the histopathological details in order to determine whether or not dysplastic lesions or carcinomas in situ were contiguous with the areas of main invasive carcinoma of each case. We immunohistologically investigated 175 sections from 35 cases which included 43 invasive carcinomas, 90 lesions of carcinoma in situ, 179 dysplasias and any remaining normal squamous epithelium adjacent to these lesions for the accumulation of mutant p53 protein.

### Immunohistochemical staining

Mouse anti-human p53 monoclonal antibody (pAb1801, Ab-2; Oncogene Science, Inc, Uniondale, NY, USA), which detects both wild and mutant types of human p53 protein, was applied to paraffin-embedded sections. For p53 immunostaining, the Histofine method (Nichirei Co. Ltd, Tokyo, Japan) was used. After dewaxing and rehydration, the antigen retrieval method using a microwave was performed. The sections were immersed in $H_2O$ and heated for 20 min at 90°C in a microwave oven, and were then cooled for 30 min at room temperature. After blocking the endogenous peroxidase activity in methanol with 0.3% hydrogen peroxide for 30 min and washing, the sections were treated with 10% normal rabbit serum for 30 min. Then the primary antibody for p53 protein was used at a working concentration of 1:100 at room temperature overnight. After washing, the sections were incubated for 30 min at room temperature with biotinylated rabbit anti-mouse immunoglobulin and peroxidase-conjugated streptaviden. Next, 0.03% 3,3′-diaminobenzidine tetrahydrochloride and 0.01% hydrogen peroxide in distilled water were used to visualize the positive areas. Methyl green was used for counterstaining.

Dark brown staining was only observed in the nucleus but not in the cytoplasm. Lesions, in which all or a majority of the cells demonstrated dark brown nuclear staining, were counted as positive. Some sections were subjected to normal serum blocking and the omission of primary antibody as negative control. Positive immnostaining was evaluated without any information on the clinical data of the patients.

### Statistical analysis.

Chi-squared tests (or Fisher's exact test when the expected number of any cell was smaller than or equal to five) were used to compare the significance of the p53 status concerning various oesophageal lesions or clinicopathological parameters.

Survival curves were then plotted with the method of Kaplan–Meier. The survivals of different groups were compared by the log-rank test.

### RESULTS

### p53 expression in various oesophageal lesions

The results of p53 expression in oesophageal squamous cell dysplasias and invasive carcinomas detected by immunostaining are shown in Figures 1 and 2 respectively. Regardless of the variety of lesions, ranging from mild dysplasia to invasive carcinoma, p53 expression was diffusely detected in the proliferative atypical nuclei of each lesion (Figures 1 and 2).

The overall frequencies of p53 expression in various oesophageal lesions are shown in Table 1. No diffuse p53 staining was detected in normal squamous epithelium specimens. On the other hand, 51.4% (92 of 179 lesions) of dysplasia, 51.0%

 © 2000 Cancer Research Campaign

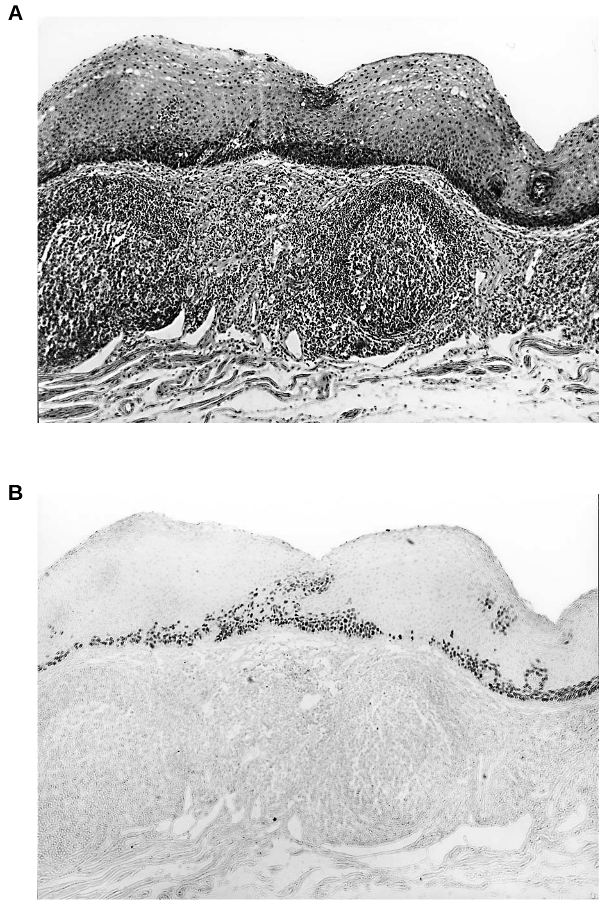

Figure 1 (A) Mild dysplasia. An atypical proliferative zone, where the mitotic activity, hyperchromatism, and nuclear variability can be seen, is limited to the basal zone. In addition, a cytoplasmic maturation is observed in a superficial zone. Beneath the dysplastic lesions and carcinoma in situ, lymphoid proliferation is frequently observed (H&E, × 100). (B) Immunohistochemical detection of p53 protein in serial sections corresponding to (A). Immunoreactivity for p53 is located in the nuclei of atypical proliferative cells in the basal zone (× 100)

(46 of 90 lesions) of carcinoma in situ, and 46.5% (20 of 43 lesions) of invasive carcinoma expressed p53. Moreover, the p53 expression for each degree of dysplasias was 59.7% (40 of 67 lesions) for mild dysplasia, 48.7% (19 of 39 lesions) for moderate dysplasia and 45.2% (33 of 73 lesions) for severe dysplasia, respectively. Therefore, as mentioned above, almost half of all lesions, from mild dysplasia to invasive carcinoma, expressed p53 protein.

### p53 expression in dysplasias associated with carcinoma

The p53 expression was next compared between dysplasias and invasive carcinomas for each case. After excluding seven cases of multiple primary invasive carcinomas with variation in the p53 expression, 28 cases were evaluated. Among the 28 invasive carcinomas observed in these 28 cases, 14 carcinomas (50.0%) diffusely expressed p53 protein. In addition, p53 expression was detected in 37 of 39 (94.8%) dysplasias contiguous to these p53-positive carcinomas, while five of 30

**Table 1** Expression of p53 in various oesophageal lesions

| | Number of lesions | Positive (%) | Negative (%) |
|---|---|---|---|
| Normal epithelium | 35 | 0 (0.0) | 35 (100.0) |
| Dysplasia | 179 | 92 (51.4) | 87 (48.6) |
| Mild dysplasia | 67 | 40 (59.7) | 27 (40.3) |
| Moderate dysplasia | 39 | 19 (48.7) | 20 (51.3) |
| Severe dysplasia | 73 | 33 (45.2) | 40 (54.8) |
| Carcinoma in situ | 90 | 46 (51.0) | 44 (49.0) |
| Invasive carcinoma | 43 | 20 (46.5) | 23 (53.5) |

**Table 2** Expression of p53 in both contiguous dysplasia and carcinoma

| Carcinoma | Dysplasias (*n*) | Contiguous dysplasia Positive (%) | Contiguous dysplasia Negative (%) |
|---|---|---|---|
| Positive (*n* = 14) | 39 | 37 (94.8) | 2 (5.2) |
| Negative (*n* = 14) | 30 | 5 (16.7) | 25 (83.3) |
| Total (*n* = 28) | 69 | 42 (60.8) | 27 (39.1) |

P < 0.0001

**Table 3** Expression of p53 in isolated dysplasia and carcinoma

| Carcinoma | Dysplasias (*n*) | Isolated dysplasia Positive (%) | Isolated dysplasia Negative (%) |
|---|---|---|---|
| Positive (*n* = 14) | 25 | 11 (44.0) | 14 (56.0) |
| Negative (*n* = 14) | 18 | 11 (61.1) | 7 (38.9) |
| Total (*n* = 28) | 43 | 22 (51.2) | 21 (48.8) |

(16.7%) dysplasias contiguous to p53-negative carcinomas expressed p53 protein. This difference was statistically significant ($P < 0.0001$) (Table 2). An example of p53 expression in one case, in which the contiguous existence of microinvasive carcinoma, carcinoma in situ, and epithelial dysplasia was recognized, is shown in Figure 3. The patterns of p53 expressions in the carcinoma in situ and dysplasia were consistent with those in invasive carcinomas that were contiguous to each other.

In contrast to the above findings, the isolated dysplasias without contiguity to the carcinomas in the p53-positive cases, only expressed p53 protein in 44.0% of lesions (11 of 25 lesions) (Table 3). These data thus suggested that the expression of p53 protein in the dysplasias depended on the degree of contiguity to the invasive carcinoma that was considered to be the main lesion in the oesophagus of each patient.

### Clinicopathological features and prognoses

Correlation of p53 expression with the clinicopathologic parameters of oesophageal squamous cell carcinomas is shown in Table 4. In univariate analyses, no significant correlation could be found between p53 expression and various clinicopathologic parameters, such as age, sex, size of tumour, location, histological grade, pT, pN, M, vascular permeation or TNM stage.

No correlation between p53 expression and prognosis was observed in patients with oesophageal squamous cell carcinoma and dysplasias (Figure 4).

© 2000 Cancer Research Campaign

**Table 4** Clinicopathological features of p53-positive and -negative groups of patients with oesophageal squamous cell carcinomas

| Parameters | p53 accumulation | | Significance |
|---|---|---|---|
| | Positive (%) | Negative (%) | |
| Patients (*n*) | 17 | 18 | |
| Mean age (years) | 59.8 ± 12.0 | 67.7 ± 11.4 | N.S. |
| Sex | | | |
| Male | 16 (94.1) | 16 (88.9) | |
| Female | 1 (5.9) | 2 (11.1) | |
| Size of tumour (cm) | 3.3 ± 1.5 | 3.6 ± 2.3 | N.S. |
| Location | | | N.S. |
| upper | 1 (5.9) | 3 (16.7) | |
| middle | 11 (64.7) | 9 (50.0) | |
| lower | 5 (29.4) | 6 (33.3) | |
| Histology | | | N.S. |
| G1 (Well) | 1 (5.9) | 3 (16.7) | |
| G2 (Moderately) | 10 (58.8) | 9 (50.0) | |
| G3 (Poorly) | 6 (35.3) | 6 (33.3) | |
| Depth of invasion | | | N.S. |
| pT1 | 11 (64.7) | 11 (61.1) | |
| pT2 | 3 (17.6) | 4 (22.2) | |
| pT3 | 2 (11.8) | 2 (11.1) | |
| pT4 | 1 (5.9) | 1 (5.6) | |
| Nodal involvement | | | N.S. |
| pN0 | 13 (76.5) | 16 (88.9) | |
| pN1 | 4 (23.5) | 2 (11.1) | |
| Distant metastases | | | N.S. |
| M0 | 17 | 18 | |
| M1 | 0 | 0 | |
| Vascular permeation | | | N.S. |
| Present | 8 (47.1) | 4 (22.2) | |
| Absent | 9 (52.9) | 14 (77.8) | |
| Stage of TNM | | | N.S. |
| Stage I | 10 (58.8) | 11 (61.1) | |
| Stage IIA | 2 (11.8) | 4 (22.2) | |
| Stage IIB | 3 (17.6) | 2 (11.1) | |
| Stage III | 2 (11.8) | 1 (5.6) | |
| Stage IV | 0 | 0 | |

A

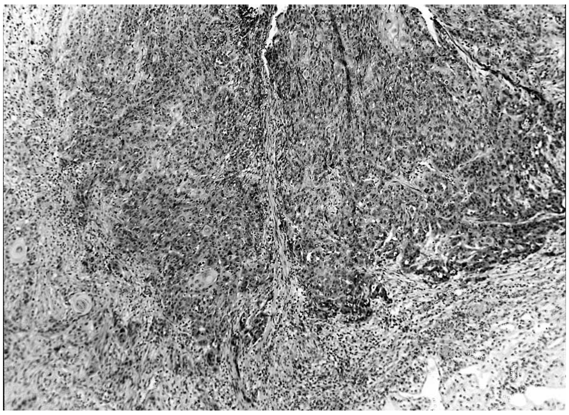

B

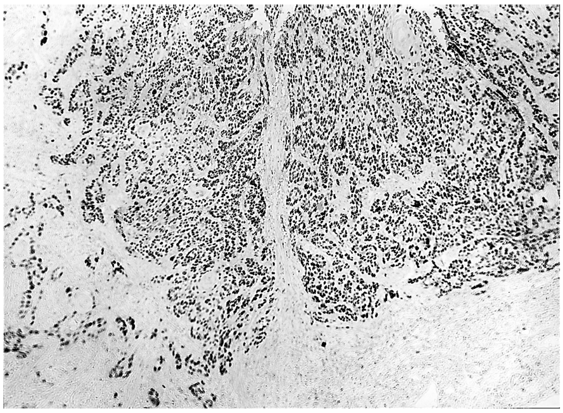

**Figure 2** (**A**) Invasive carcinoma. Moderately differentiated squamous cell carcinoma with invasion to the deeper portion of the submucosal layer (H&E, × 90). (**B**) Immunohistochemical detection of p53 protein. Immunoreactivity for p53 is diffusely detected in the nuclei of atypical proliferative cells (× 90)

### DISCUSSION

In the current study, the p53 status in oesophageal squamous cell carcinoma and dysplasia was investigated from the viewpoint of the continuity of both lesions. The p53 expression in more than half of all dysplasias apart from the p53-positive carcinomas was inconsistent with that in the carcinoma. In addition, the p53 positive rate in these isolated dysplasias was similar to the overall frequency of expression in dysplasia or carcinoma. Although the biological nature of dysplasia based on the p53 status might share strong similarity with that of carcinoma (Wang et al, 1993; Shi et al, 1999), the origin of the carcinoma and the isolated dysplasias with discordant p53 immunostaining might be different even in the individual oesophagus. These findings support the simultaneous multifocal origin of oesophageal cancer, and demonstrate the pattern of p53 alteration in the multicentric or field carcinogenesis in the oesophagus. Next, we assessed the consistency of the p53 positivity in dysplasias with that in carcinoma contiguous to each other, and as a result, 37 of 39 (94.8%) dysplasias contiguous to p53-positive carcinomas also expressed p53 protein. The p53 positivity in carcinomas and dysplasias that were contiguous to each other showed a close correlation. Although identical p53 mutations in carcinoma and nearby dysplasia have been already reported (Shi et al, 1999), our findings revealed inconsistent and multifocal origin of p53 alterations which would contribute to elucidate the mechanism of multicentric or field carcinogenesis.

Within the limits of the pre-invasive area of the oesophageal cancer, intratumoural heterogeneity of p53 gene mutation was recognized (Kawabara et al, 1998). This heterogeneity could be explained in terms of a collision of multiple clones with different p53 mutations and/or additional p53 mutations within a single tumour after clonal expansion. Our previous reports regarding serial histopathological examinations have shown that the pre-invasive lesions observed as an intra-epithelial spread was more frequently seen in the cases with less advanced cancer (Kuwano et al, 1987; 1988; Tsutsui et al, 1995). We thus speculated that the intra-epithelial carcinoma might originate from multifocal or field carcinomatous transformation rather than from an intra-epithelial spread followed by a progression of the main lesion. As a result, the intratumoural heterogeneity of p53 gene mutation in pre-invasive carcinoma is presumed to be due to the collision of multiple clones. In other words, independent and multifocal epithelial dysplastic lesions can arise simultaneously with or without various p53 alterations, some of which may develop and progress to cancer without any additional p53 alteration.

Although p53 alterations play an important role in oesophageal carcinogenesis, its role in the invasion and metastasis of

*© 2000 Cancer Research Campaign*

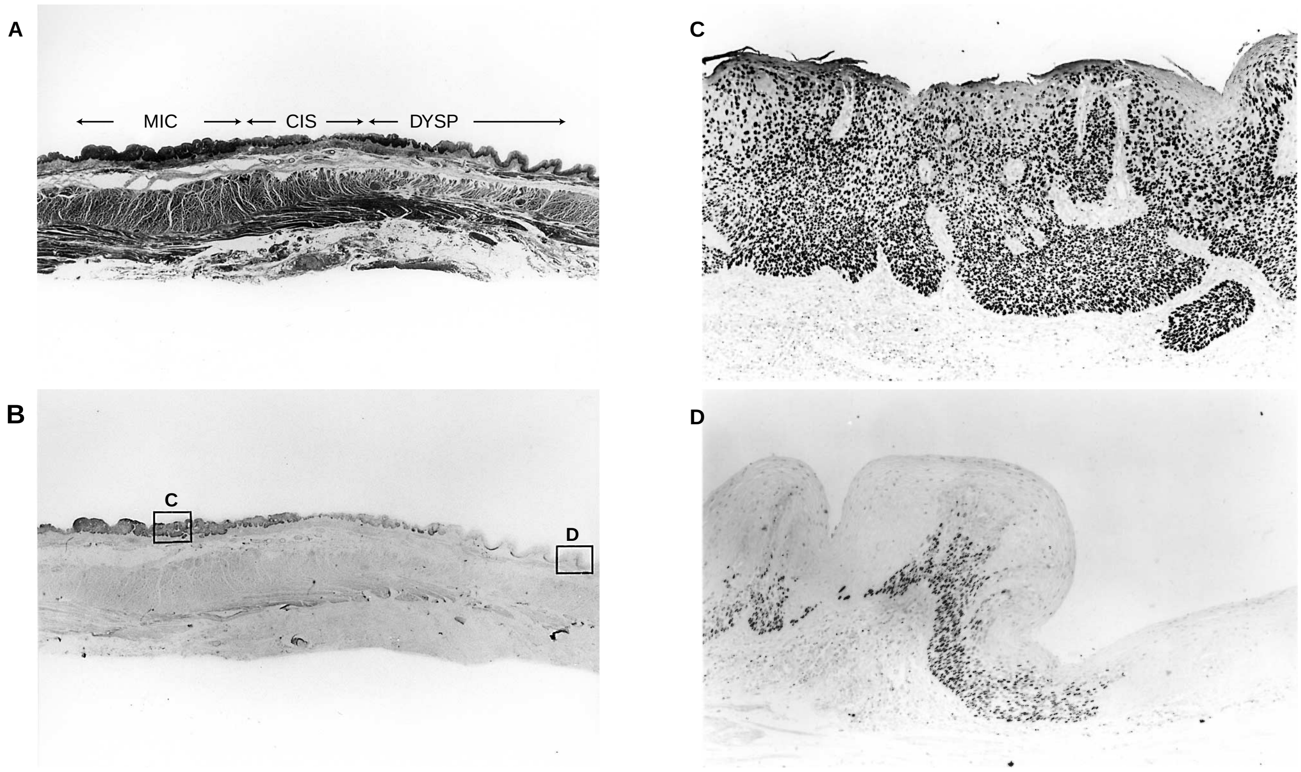

**Figure 3** (**A**) The contiguous existence of microinvasive carcinoma (MIC), carcinoma in situ (CIS), and epithelial dysplasia (DYSP) in a resected specimen of the oesophagus (H&E, × 7). (**B**) Immunohistochemical staining of p53 protein in a serial section (× 7). In this specimen, from microinvasive carcinoma (**C**) to contiguous epithelial dysplasia (**D**), the nuclei of the atypical proliferative cells all presented homogenous immunoreactivity for p53 (**C**, **D** × 70)

oesophageal carcinomas remains unclear. There have recently been many reports concerning the prognostic value of the p53 alterations in oesophageal carcinoma (Sarbia et al, 1994; Lam et al, 1997; Ikeda et al, 1999; Kanamoto et al, 1999; Kobayashi et al, 1999; Shimada et al, 1999; Wang et al, 1999). However, the reported results are inconsistent. In this study, no significant correlations were found between p53 staining and the clinicopathological features or patients survival. The prognoses demonstrated in this study were better than those in other articles, because the selected patients had been restricted to a rather earlier stage of oesophageal cancer without any preoperative treatment. Moreover, all patients analysed in this study had carcinomas coexistent with dysplasias, which were more frequently seen in the less advanced oesophageal cancer (Kuwano et al, 1993). As mentioned above, the p53 alteration in such patients might occur in the early stages of oesophageal carcinogenesis but not in stages of tumour development or progression, thus resulting in no correlation in the prognoses. Several studies have emphasized a worse prognosis in stage II or III oesophageal cancers with p53 mutations (Lam et al, 1997, Kobayashi et al, 1999). We cannot exclude the possibility that the p53 mutations may induce a growth capability to tumour cells in relatively advanced oesophageal cancer and may thus be associated with a lower patient survival.

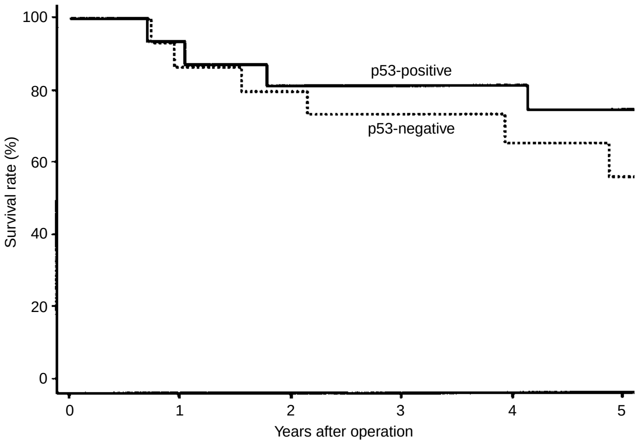

**Figure 4** Kaplan–Meier survival curves of 35 patients with oesophageal carcinoma grouped according to the p53 expression. No significant difference was found between the p53-positive group (*n* = 17) and the p53-negative group (*n* = 18) by the log-rank test

In general, the immunohistochemical approach for detecting p53 alterations must be viewed with care. We cannot rule out the possibility of non-mutational mechanisms for p53 protein accumulation, such as a stabilization of the wild-type p53 protein (Finlay et al, 1988; van den Heuvel et al, 1990) or an inactivation of the enzyme for p53 protein degradation (Ciechanover et al, 1991). In addition, negative p53 immunostaining does not necessarily rule out p53 mutations, because allelic loss or frameshift mutations may not cause any protein accumulation. Nevertheless, the method of p53 immunostaining is strongly suggested to be useful in this situation to analyse the p53 inconsistency in the cancerization field of the individual oesophagus.

In conclusion, the multifocal p53 alterations seen in multicentric cancerous and precancerous lesions could demonstrate further evidence for field carcinogenesis of the human oesophagus.

© 2000 Cancer Research Campaign

Therefore, a careful follow-up by frequent biopsies using endoscopic Lugol staining test (Mori et al, 1993) is needed for early detection of cancer development from these dysplastic lesions, while it is also important to pay close attention to the multicentric occurrence of these lesions. Although dysplasia should not be considered 'clinical cancer' since it stays in the epithelium without invasion, the biological nature of dysplasia may be similar to that of carcinoma regarding p53 expression. An endoscopic mucosal resection is thus indicated for both an accurate diagnosis and a good curative effect, when a p53 abnormality is detected and/or a diagnosis of carcinoma cannot be ruled out based on the findings of biopsy specimens.

### ACKNOWLEDGEMENT

We thank Brian T Quinn for comments on the manuscript.

### REFERENCES

Bennett WP, Hollstein MC, He A, Zhu SM, Resau J, Trump BF, Metcalf RA, Welsh JA, Midgley C, Lane DP and Harris CC (1991) Archival analysis of p53 genetic and protein alterations in Chinese esophageal cancer. *Oncogene* **6**: 1779–1784

Bennett WP, Hollstein MC, Metcalf RA, Welsh JA, He A, Zhu SM, Kusters I, Resau JH, Trump BF, Lane DP and Harris CC (1992) p53 mutation and protein accumulation during multistage human esophageal carcinogenesis. *Cancer Res* **52**: 6092–6097

Ciechanover A, DiGiuseppe JA, Bercovich B, Orian A, Richter JD, Schwartz AL and Brodeur GM (1991) Degradation of nuclear oncoproteins by the ubiquitin system in vitro. *Proc Natl Acad Sci USA* **88**: 139–143

Finlay CA, Hinds PW, Tan TH, Eliyahu D, Oren M and Levine AJ (1988) Activating mutations for transformation by p53 produce a gene product that forms an hsc70–p53 complex with an altered half-life. *Mol Cell Biol* **8**: 531–539

Gao H, Wang LD, Zhou Q, Hong JY, Huang TY and Yang CS (1994) p53 tumour suppressor gene mutation in early esophageal precancerous lesions and carcinoma among high-risk populations in Henan, China. *Cancer Res* **54**: 4342–4346

Hall PA and Lane DP (1994) p53 in tumour pathology: can we trust immunohistochemistry? revisited. *J Pathol* **172**: 1–4

Hollstein M, Sidransky D, Vogelstein B and Harris CC (1991) p53 mutations in human cancers. *Science* **253**: 49–53

Ikeda G, Isaji S, Chandra B, Watanabe M and Kawarada Y (1999) Prognostic significance of biologic factors in squamous cell carcinoma of the esophagus. *Cancer* **86**: 1396–1405

Kanamoto A, Kato H, Tachimori Y, Watanabe H, Nakanishi Y, Kondo H, Yamaguchi H, Gotoda T, Muro K and Matsumura Y (1999) No prognostic significance of p53 expression in esophageal squamous cell carcinoma. *J Surg Oncol*. **72**: 94–98

Kawabara S, Ajioka Y, Watanabe H, Hitomi J, Nishikura K and Hatakeyama K (1998) Heterogeneity of p53 mutational status in esophageal squamous cell carcinoma. *Jpn J Cancer Res* **89**: 405–410

Kobayashi S, Koide Y, Endo M, Isono K and Ochiai T (1999) The p53 gene mutation is of prognostic value in esophageal squamous cell carcinoma patients in unified stages of curability. *Am J Surg* **177**: 497–502

Koga Y, Sugimachi K, Kuwano H, Mori M and Matsufuji H (1988) Cytophotometric DNA analysis of esophageal dysplasia and carcinoma induced in rats by N-methyl-N-amylnitrosamine. *Eur J Cancer Clin Oncol* **24**: 643–651

Koga Y, Kuwano H and Sugimachi K (1996) Biologic characteristics of esophageal epithelial dysplasia assessed by proliferating cell nuclear antigen. *Cancer* **77**: 237–244

Kuwano H, Matsuda H, Matsuoka H, Kai H, Okudaira Y and Sugimachi K (1987) Intra-epithelial carcinoma concomitant with esophageal squamous cell carcinoma. *Cancer* **59**: 783–787

Kuwano H, Nagamatsu M, Ohno S, Matsuda H, Mori M and Sugimachi K (1988) Coexistence of intraepithelial carcinoma and glandular differentiation in esophageal squamous cell carcinoma. *Cancer* **62**: 1568–1572

Kuwano H, Watanabe M, Sadanaga N, Ikebe M, Mori M and Sugimachi K (1993) Squamous epithelial dysplasia associated with squamous cell carcinoma of the esophagus. *Cancer Lett* **72**: 141–147

Lam KY, Tsao SW, Zhang D, Law S, He D, Ma L and Wong J (1997) Prevalence and predictive value of p53 mutation in patients with oesophageal squamous cell carcinomas: a prospective clinico-pathological study and survival analysis of 70 patients. *Int J Cancer* **74**: 212–219

Mori M, Adachi Y, Matsushima T, Matsuda H, Kuwano H and Sugimachi K (1993) Lugol staining pattern and histology of esophageal lesions. *Am J Gastroenterol* **88**: 701–705

Morita M, Kuwano H, Yasuda M, Watanabe M, Ohno S, Saito T, Furusawa M and Sugimachi K (1994) The multicentric occurrence of squamous epithelial dysplasia and squamous cell carcinoma in the esophagus. *Cancer* **74**: 2889–2895

Nagamatsu M, Mori M, Kuwano H, Sugimachi K and Akiyoshi T (1992) Serial histologic investigation of squamous epithelial dysplasia associated with carcinoma of the esophagus. *Cancer* **69**: 1094–1098

Rogel A, Popliker M, Webb CG and Oren M (1985) p53 cellular tumour antigen: Analysis of mRNA levels in normal adult tissues, embryos and tumours. *Mol Cell Biol* **5**: 2851–2855

Sarbia M, Porschen R, Borchard F, Horstmann O, Willers R and Gabbert HE (1994) p53 protein expression and prognosis in squamous cell carcinoma of the esophagus. *Cancer* **74**: 2218–2223

Shi ST, Yang GY, Wang LD, Xue Z, Feng B, Ding W, Xing EP and Yang CS (1999) Role of p53 gene mutations in human esophageal carcinogenesis: results from immunohistochemical and mutation analyses of carcinomas and nearby noncancerous lesions. *Carcinogenesis* **20**: 591–597

Shimada Y, Imamura M, Watanabe G, Uchida S, Harada H, Makino T and Kano M (1999) Prognostic factors of oesophageal squamous cell carcinoma from the perspective of molecular biology. *Br J Cancer* **80**: 1281–1288

Sobin LH and Wittekind CH (1997) *TNM Classification of Malignant Tumours, 5th edn*. Wiley-Liss: New York

Tsutsui S, Kuwano H, Yasuda M, Nozoe T, Watanabe M, Kitamura M and Sugimachi K (1995) Extensive spreading carcinoma of the esophagus with invasion restricted to the submucosa. *Am J Gastroenterol* **90**: 308–313

van den Heuvel SJL, van Laar T, Kast WM, Melief CJM, Zantema A and van der Eb AJ (1990) Association between the cellular p53 and the adenovirus 5 E1B-55kd proteins reduces the oncogenicity of Ad-transformed cells. *EMBO J* **8**: 2621–2629

Vijeyasingam R, Darnton SJ, Jenner K, Allen CA, Billingham C and Matthews HR (1994) Expression of p53 protein in oesophageal carcinoma: clinicopathological correlation and prognostic significance. *Br J Surg* **81**: 1623–1626

Wagata T, Shibagaki I, Imamura M, Shimada Y, Toguchida J, Yandell DW, Ikenaga M, Tobe T and Ishizaki K (1993) Loss of 17p, mutation of p53 gene, and overexpression of p53 protein in esophageal squamous cell carcinomas. *Cancer Res* **53**: 846–850

Wang LD, Hong JY, Qiu SL, Gao H and Yang CS (1993) Accumulation of p53 protein in human esophageal precancerous lesions: a possible early biomarker for carcinogenesis. *Cancer Res* **53**: 1783–1787

Wang LD, Zhou Q, Hong JY, Qiu SL and Yang CS (1996) p53 protein accumulation and gene mutations in multifocal esophageal precancerous lesions from symptom free subjects in a high incidence area for esophageal carcinoma in Henan, China. *Cancer* **77**: 1244–1249

Wang LS, Chow KC, Chi KH, Liu CC, Li WY, Chiu JH and Huang MH (1999) Prognosis of esophageal squamous cell carcinoma: analysis of clinicopathological and biological factors. *Am J Gastroenterol* **94**: 1933–1940

Watanabe H, Jass JR and Solin L (1990) *Histological typing of oesophageal and gastric tumours 2nd edn.* pp 11–18. Springer-Verlag: Berlin, Heidelberg, New York, London, Paris, Tokyo, Hong Kong

 *© 2000 Cancer Research Campaign*
